# Propensity score matching analysis of the association between *Helicobacter pylori* infection and colorectal polyps: A case-control study

**DOI:** 10.1097/MD.0000000000048957

**Published:** 2026-05-22

**Authors:** Yating Gong, Zhen Fan

**Affiliations:** a Department of Gastroenterology, The First People’s Hosptial of Fuyang, Hangzhou, Zhejiang, China; b Department of Gastroenterology, Hangzhou First People’s Hospital, Hangzhou, Zhejiang, China.

**Keywords:** association, colorectal polyps, *Helicobacter pylori*, propensity score matching

## Abstract

The association between *Helicobacter pylori* (*H. pylori*) infection and colorectal polyps remains controversial. This study was a retrospective observational study. A total of 1106 patients who underwent gastrointestinal endoscopy at the First People’s Hospital of Fuyang from January 2021 to May 2023 were enrolled. Based on colonoscopic findings, patients were stratified into a colorectal polyp group (n = 401) and a control group (n = 705). Propensity score matching (PSM) was applied in a 1:1 ratio to balance baseline confounders. Conditional logistic regression evaluated the association between *H. pylori* infection and colorectal polyps. Before PSM, significant differences were observed in body mass index, age, male sex, alcohol consumption history, smoking history, diabetes mellitus, and hypertension between the colorectal polyp group and control group (*P* < .05). After PSM, 347 matched pairs were generated. Using the matched sample, conditional logistic regression analysis identified *H. pylori* infection as a risk factor for colorectal polyps (odds ratio = 1.65, 95% confidence interval [CI]: 1.17–2.33; *P* = .004). Receiver operating characteristic curve analysis yielded an area under the curve of 0.549 (95% CI: 0.506–0.592; *P* = .025) for *H. pylori* infection in predicting polyps. The application of PSM further validates the association between *H. pylori* infection and colorectal polyps. *H. pylori* infection alone demonstrates limited predictive utility for colorectal polyp risk.

## 1. Introduction

The incidence of colorectal polyps has risen markedly in China in recent years.^[1]^ Approximately 75% of colorectal cancers (CRCs) originate from adenomatous polyps.^[2]^ CRC can also develop from hyperplastic polyps.^[3,4]^ Therefore, increasing the detection and treatment of polyps reduces the incidence of CRC. While colonoscopy remains the gold standard for polyp detection, its widespread adoption is limited by high costs, burdensome bowel preparation, and patient reluctance. Therefore, risk stratification to identify high-risk populations for targeted colonoscopic screening represents an effective strategy for CRC prevention.

Some studies^[5–7]^ have shown that Helicobacter pylori (*H. pylori*) infection promotes the development of colorectal polyps, whereas others^[8]^ have reported no significant increase in polyp incidence associated with the infection. Thus, the correlation between *H. pylori* infection and colorectal polyps remains controversial. Notably, China has a high *H. pylori* infection rate, estimated at 55 to 60%.^[9]^ Clarifying the association between colorectal polyps and *H. pylori* infection is therefore of critical importance.

Multiple factors contribute to the development of colorectal polyps, and traditional statistical methods have been widely used to analyze these factors. However, when numerous confounding factors are present, traditional analytical approaches often fail to yield reliable results because of significant baseline differences between groups in retrospective studies. Additionally, randomized controlled trials are costly and time-intensive. In propensity score matching (PSM), individuals in the observation and control groups are matched based on their propensity scores. For each individual in the observation group, one or more control individuals with identical or similar scores are selected; those who cannot be successfully matched are excluded. This process balances the distribution of confounding factors between the 2 groups after matching, thereby minimizing bias and ensuring comparability.^[10]^ This method has rarely been reported in previous studies on this topic. In this study, PSM was used to assess the association between colorectal polyp development and *H. pylori* infection with enhanced accuracy and cost-effectiveness. These findings provide an evidence-based foundation for the clinical prevention and treatment of colorectal polyps.

## 2. Materials and methods

### 2.1. Patient selection

No studies using PSM to examine the association between *H. pylori* and colorectal polyps are available for direct reference. Accordingly, sample size was estimated based on the exposure rates reported by Ding H et al.^[11]^ Sample size was estimated using the case-control study formula (α = 0.05, power = 80%). Considering the potential for sample loss during PSM, we increased the required sample size by 30%. To ensure sufficient statistical power after matching, we inflated the target sample size by 30% to 766 patients.

**Figure 1.**
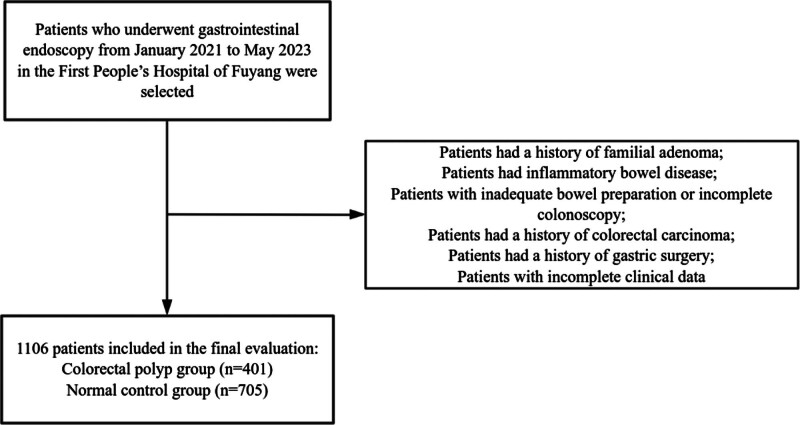
The study flow chart. Using predefined inclusion and exclusion criteria, 1106 patients were ultimately enrolled in the study.

One thousand one hundred and 6 patients who underwent gastrointestinal endoscopy from January 2021 to May 2023 in the First People’s Hospital of Fuyang were enrolled (Fig. 1). The inclusion criteria were as follows: age ≥ 18 years; following complete colonoscopy, polypoid lesions were pathologically diagnosed as colorectal polyps, with their histological types recorded and categorized as adenomatous or non-adenomatous; and diagnosis of *H. pylori* infection by microscopic staining of gastric mucosal tissue sections. The exclusion criteria were as follows: a history of familial adenoma; presence of inflammatory bowel disease; no total colonoscopy; inadequate bowel preparation; a history of colorectal carcinoma; history of gastric surgery; and incomplete clinical data. Based on colonoscopy results, eligible cases were divided into 2 groups: the colorectal polyp group (n = 401) and the normal control group (n = 705). This study protocol was approved by the First People’s Hospital of Fuyang (2023-LW-043), and our research adhered to the principles of the Declaration of Helsinki. The requirement for informed consent was waived owing to the retrospective nature of this study.

### 2.2. Data collection

Basic demographic and clinical data were collected, including sex, age, and body mass index (BMI), etc.

Smoking history: Defined as active smoking for >1 year with a minimum intake of 1 cigarette/day.

Alcohol consumption: Defined as regular intake for >1 year with ≥1 drink/week.

Diabetes mellitus: Diagnosed if any of the following were met:

(1) Prior clinical diagnosis of diabetes;
(2) Fasting plasma glucose ≥ 7.0 mmol/L;
(3) Presence of classic symptoms and random plasma glucose ≥ 11.1 mmol/L.

Hypertension: Diagnosed if either:

(1) Documented clinical diagnosis of hypertension;
(2) Systolic blood pressure ≥ 140 mm Hg and/or diastolic blood pressure ≥ 90 mm Hg on a non-same day.

All patients underwent bowel preparation before colonoscopy. Both gastroscopy and colonoscopy were performed using an Olympus 290. During gastroscopy, gastric mucosal tissue was routinely collected for pathological detection of *H. pylori*. During colonoscopy, the scope was advanced to the terminal ileum; the size and number of polypoid lesions were recorded, and pathological biopsies were obtained. Patients with colorectal polyps were stratified into 2 groups based on the size of the largest polyp: those with polyps ≥1 cm and <1 cm in maximum diameter. According to polyp number, cases were classified as single (1 polyp) or multiple (≥2 polyps). Polyps were histologically categorized as adenomatous or non-adenomatous. To ensure data integrity, we conducted a complete-case analysis, excluding patients with any missing covariate values. Consequently, the final cohort had no missing data for any variables.

### 2.3. Statistical analyses

Normally distributed data were expressed as mean ± standard deviation and compared using an independent samples *t*-test, while non-normally distributed data were presented as median (interquartile range) and analyzed using the Mann–Whitney *U* test. Count data were expressed as numbers (percentages) and compared using the chi-square test. Patients were stratified into a colorectal polyp group and a normal control group. A 1:1 matched analysis was performed using nearest-neighbor matching with a caliper of 0.05. Covariates with an absolute standardized mean difference (SMD) < 0.1 were considered well balanced, and balance was visualized using a Love plot. Conditional logistic regression was used to evaluate the association between *H. pylori* infection and colorectal polyps, and the area under the receiver operating characteristic curve was calculated to assess its predictive capability. Sensitivity analysis for unmeasured confounding was conducted using the *E*-value method (VanderWeele & Ding, 2017). The variance inflation factor (VIF) was used to assess multicollinearity among the independent variables. A VIF value of <5 indicated no severe multicollinearity. Multivariate analysis of variance was used to examine the interaction between *H. pylori* infection and clinical characteristics of colorectal polyps. Analyses were performed in R version 4.3.2 (R Foundation for Statistical Computing, Vienna, Austria) using two-tailed tests (α = 0.05); *P* < .05 defined statistical significance.

## 3. Result

### 3.1. Patients’ baseline characteristics

The study finally included 1106 patients who met the inclusion and exclusion criteria. Before PSM, the BMI (*P* < .05), age (*P* < .05), male (*P* < .05), alcohol consumption (*P* < .05), smoking history (*P* < .05), diabetes mellitus (*P* < .05), and hypertension (*P* = .011) at admission were significantly higher in the colorectal polyp group than in the normal control group (Table 1).

**Table 1:** Comparison of baseline characteristics between colorectal polyp group and normal control group before PSM.

Characteristics		Colorectal polyp group (n = 401)		Normal control group (n = 705)	*P* value	SMD
BMI	24.24 ± 3.07			23.10 ± 2.97	<.05	0.37
Age	56.00 (49.00, 63.00)			51.00 (41.00, 59.00)	<.05	0.57
*H. pylori* infection	131 (32.67%)			157 (22.27%)	<.05	0.22
Female	161 (40.15%)			415 (58.87%)	<.05	0.38
Alcohol consumption			75 (18.70%)	76 (10.78%)	<.05	0.20
Smoking history			108 (26.93%)	122 (17.30%)	<.05	0.22
Hypertension			113 (28.18%)	151 (21.42%)	.011	0.15
Diabetes mellitus			40 (9.98%)	33 (4.68%)	<.05	0.18

BMI = body mass index, *H. pylori* = *Helicobacter pylori*, PSM = propensity score matching.

A total of 347 pairs were matched using the 1:1 nearest-neighbor matching method with a caliper of 0.05. After matching, there was no statistically significant difference between the 2 groups in terms of BMI, age, male, alcohol consumption, smoking history, diabetes mellitus, and hypertension, indicating that these baseline variables were well balanced between the groups (Table 2).

**Table 2:** Comparison of baseline characteristics between colorectal polyp group and normal control group after PSM.

Characteristics	Colorectal polyp group (n = 347)	Normal control group (n = 347)	*P* value	SMD
BMI	23.97 ± 3.02	23.71 ± 2.89	.255	0.08
Age	55.00 (48.00, 61.00)	56.00 (48.50, 63.00)	.262	0.05
*H. pylori* infection	112 (32.28%)	78 (22.48%)	.004	0.21
Female	157 (45.24%)	163 (46.97%)	.648	0.04
Alcohol consumption	50 (14.41%)	52 (14.99%)	.830	0.02
Smoking history	82 (23.63%)	80 (23.05%)	.858	0.01
Hypertension	91 (26.22%)	87 (25.07%)	.728	0.03
Diabetes mellitus	27 (7.78%)	28 (8.07%)	.888	0.01

BMI = body mass index, *H. pylori* = *Helicobacter pylori*, PSM = propensity score matching.

### 3.2. Covariate balance before and after PSM using SMD

**Figure 2.**
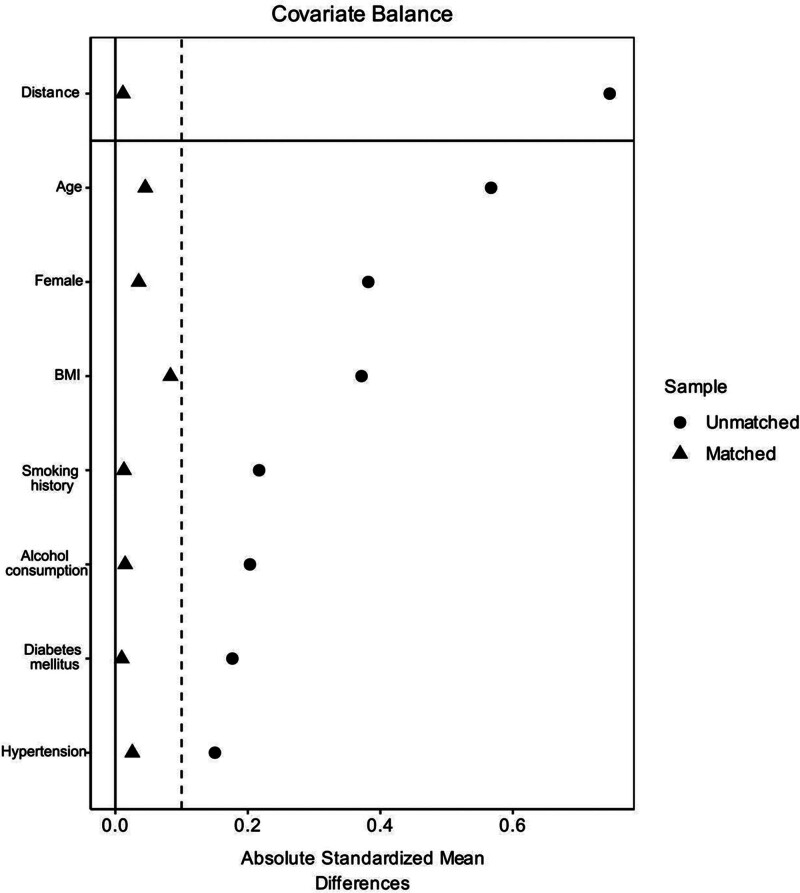
Love plot showing covariate balance before and after PSM. Circles (●) represent SMDs before matching, and triangles (▲) represent SMDs after matching. Vertical dashed lines indicate the prespecified threshold for acceptable balance (SMD = 0.1). SMD values closer to zero indicate better balance between treatment groups. PSM = propensity score matching, SMD = standardized mean difference.

Before matching, the SMD for each covariate was >0.1. After matching, the SMD for all covariates was <0.1, indicating that good balance was successfully achieved between the 2 groups (Fig. 2).

### 3.3. Association between H. pylori infection and colorectal polyps: a conditional logistic regression analysis

Univariate conditional logistic regression analysis indicated an association between *H. pylori* infection and colorectal polyps (odds ratio [OR] = 1.65, 95% confidence interval [CI]: 1.17–2.33; *P* < .05) (Table 3). Furthermore, multivariate conditional logistic regression confirmed *H. pylori* infection as an independent risk factor for colorectal polyps (OR = 1.65, 95% CI: 1.17–2.33; *P* = .004) (Table 4).

**Table 3:** Univariate conditional logistic regression analysis for association between *H. pylori* infection and colorectal polyps.

Characteristics	OR	95% CI	*P* value
BMI	1.04	0.98–1.10	.21
Age	0.99	0.98–1.01	.46
*H. pylori* infection	1.65	1.17–2.33	<.05
Female	1.09	0.78–1.51	.61
Alcohol consumption	0.95	0.62–1.47	.83
Smoking history	1.03	0.72–1.48	.86
Hypertension	1.07	0.74–1.56	.71
Diabetes mellitus	0.96	0.54–1.70	.88

BMI-body mass index; CI = confidence interval, *H. pylori*-*Helicobacter pylori*, OR = odds ratio.

**Table 4:** Multivariate conditional logistic regression analysis for association between *H. pylori* infection and colorectal polyps.

Characteristics	OR	95% CI	*P* value
*H. pylori* infection	1.65	1.17–2.33	.004

CI = confidence interval, *H. pylori* = *Helicobacter pylori*.

### 3.4. Sensitivity analysis for unmeasured confounding using the E-value

In sensitivity analysis for unmeasured confounding, the *E*-value for the point estimate was 2.69, and the *E*-value for the 95% CI lower limit was 1.62. These results indicated that moderate unmeasured confounding would be required to explain away our findings.

### 3.5. Assessment of multicollinearity among independent variables using the VIF

The VIF values for all covariates were below the conventional threshold of 5. These results indicated no significant multicollinearity (Table 5).

**Table 5:** Assessment of multicollinearity among independent variables using the VIF.

Characteristics	VIF
BMI	1.11
Age	1.18
*H. pylori* infection	1.01
Female	1.45
Alcohol consumption	1.20
Smoking history	1.42
Hypertension	1.22
Diabetes mellitus	1.04

BMI = body mass index, *H. pylori* = *Helicobacter pylori*, VIF = variance inflation factor.

### 3.6. Value of H. pylori infection for predicting colorectal polyps

**Figure 3.**
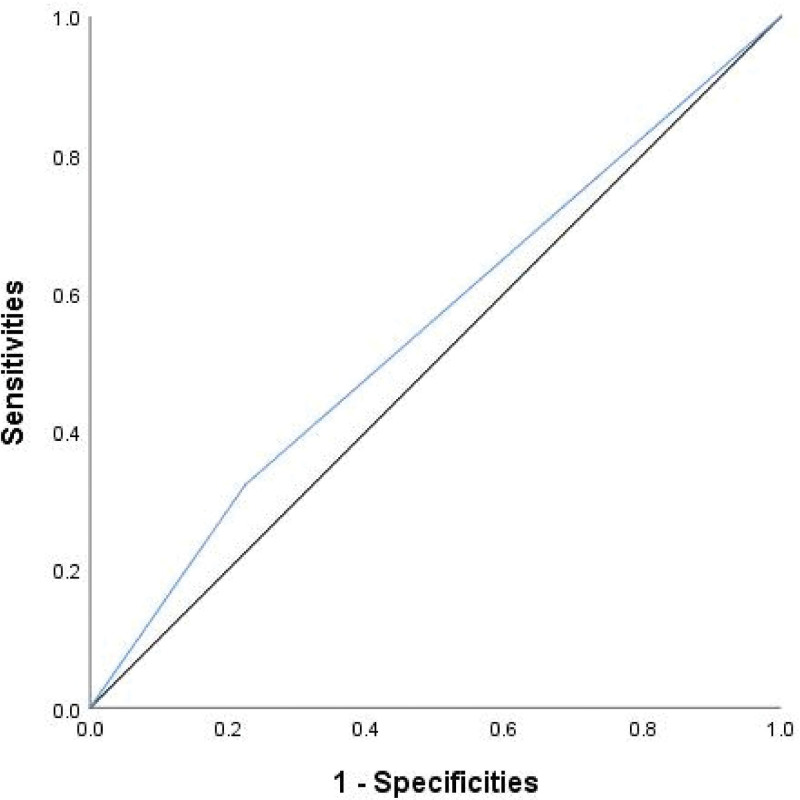
The ROC curve for *H. pylori* infection in predicting colorectal polyps demonstrated an AUC of 0.549. AUC = area under the curve, *H. Pylori* = *Helicobacter pylori*, ROC = receiver operating characteristic.

Receiver operating characteristic curve analysis demonstrated that the area under the curve (AUC) for predicting colorectal polyps using *H. pylori* infection was 0.549 (95% CI: 0.506–0.592; *P* = .025) (Fig. 3).

### 3.7. Association between H. pylori infection and histological subtypes of colorectal polyps

Among patients with colorectal polyps, *H. pylori* positivity rates were comparable between non-adenomatous (144 cases; 31.94%) and adenomatous polyps (203 cases; 32.51%). Multivariable logistic regression analysis revealed no significant association between *H. pylori* infection and colorectal polyp pathology (OR = 1.09, 95% CI: 0.68–1.76; *P* = .716) (Table 6).

**Table 6:** Association of histological subtype, number, and diameter of colorectal polyps with *H. pylori* infection.

*H. pylori* infection	Histological subtypes		Number		Diameter	
	Non-adenomatous polyps	Adenomatous polyps	Single polyps	Multiple polyps	<1.0 cm	≥1.0 cm
	46 (31.94%)	66 (32.51%)	60 (30.61%)	52 (34.44%)	101 (31.37%)	11 (44.00%)
OR	1.09		1.28		1.80	
95% CI	0.68–1.76		0.79–2.06		0.77–4.19	
*P* value	.716		.312		.173	

CI = confidence interval, *H. pylori = Helicobacter pylori*, OR = odds ratio.

### 3.8. Association of colorectal polyp multiplicity with H. pylori infection

In the colorectal polyp group, 196 patients had single polyps with an *H. pylori*-positive rate of 30.61%, and 151 patients had multiple polyps with an *H. pylori*-positive rate of 34.44%, which was higher than that of the single polyp group. Multifactorial logistic regression analysis showed that the association between *H. pylori* infection and the number of colorectal polyps was not statistically significant (OR = 1.28, 95% CI: 0.79–2.06, *P* = .312) (Table 6).

### 3.9. Association between H. pylori infection and colorectal polyp diameter

Among patients with colorectal polyps, 322 had polyps < 1.0 cm with an *H. pylori*-positive rate of 31.37%, and 25 had polyps ≥ 1.0 cm with an *H. pylori*-positive rate of 44.00%, which was higher than that in the <1.0 cm group. Multivariable logistic regression analysis showed that the association between *H. pylori* infection and colorectal polyp size was not statistically significant (OR = 1.80, 95% CI: 0.77–4.19, *P* = .173) (Table 6).

## 4. Discussion

*H*. *pylori* colonizes the gastric mucosa and is recognized as a Class I carcinogen for gastric cancer. Recent studies^[12]^ found that *H. pylori* may contribute to extra-gastric diseases, including colorectal cancer, inflammatory bowel disease, and pancreatic cancer, through mechanisms such as chronic inflammation, toxin release, and gut microbiota dysbiosis; however, these associations remain inconclusive. Most patients with colorectal polyps are asymptomatic, and progression to CRC typically takes 10 to 15 years.^[13]^ The incidence of colorectal polyps and cancer has risen steadily in recent decades. Thus, clarifying the relationship between *H. pylori* infection and the development of colorectal polyps is crucial for designing targeted interventions to prevent CRC.

This study revealed a significantly higher prevalence of *H. pylori* infection in the colorectal polyp group compared to the control group. After adjusting for confounders using PSM, conditional logistic regression analysis identified *H. pylori* infection as an independent risk factor for colorectal polyps, OR = 1.65 (95% CI: 1.17–2.33, *P* = .004). Wang M et al^[14]^ demonstrated that *H. pylori*-infected individuals had a 2.19-fold higher risk of colorectal polyps compared to uninfected individuals. Similarly, Zhao XX et al^[15]^ reported an OR of 2.68 for colorectal polyps in *H. pylori*-infected individuals. However, these estimates were derived from traditional statistical analyses without confounder adjustment through matching, which may have led to an overestimation of the association. In contrast, our study found a 1.65-fold higher risk of colorectal polyps in *H. pylori*-infected individuals compared to uninfected individuals, suggesting a weaker association than reported in prior studies. The PSM approach provided a more rigorous assessment of the relationship between *H. pylori* infection and colorectal polyps. As is well known, an AUC of 0.5 indicates that a diagnostic method has no discriminatory power, whilst an AUC between 0.5 and 0.7 indicates weak discriminatory power. In this study, the AUC for predicting colorectal polyps based on *H. pylori* infection was 0.549, suggesting that *H. pylori* infection alone is of limited value in predicting colorectal polyp risk.

We did not formally test for interaction terms. Previous research has shown that omitting interaction terms from propensity score models does not necessarily introduce bias, particularly when post-matching covariate balance is achieved.^[16]^ In our analysis, post-matching balance diagnostics demonstrated good covariate balance, with all SMDs below 0.1, indicating that a main-effects model was sufficient for creating comparable groups.

Colorectal polyps are lesions caused by multiple factors. Univariate analysis in this study revealed statistically significant associations of age, obesity, and sex with colorectal polyp occurrence, consistent with previous reports.^[17–20]^ A key strength of this study lies in the use of PSM to control for confounding factors. By matching individuals in the colorectal polyp group and the normal control group according to their propensity scores, the distribution of confounding factors (such as smoking history, hypertension, and diabetes mellitus) between the groups is balanced, thereby reducing bias in this retrospective study. This approach is more capable of validating the relationship between *H. pylori* infection and colorectal polyps, which has been rarely reported in the previous literature. Furthermore, all patients underwent gastroscopy and colonoscopy, eliminating temporal bias and improving the reliability of the analysis.

In our study, *H. pylori* infection rates were higher in adenomatous polyps than in non-adenomatous polyps, in patients with multiple polyps than in those with a single polyp, and in polyps ≥ 1.0 cm than in those <1.0 cm; however, none of these differences reached statistical significance. These findings align with those of Brim H et al.^[6]^ Similarly, Wang M et al^[14]^ reported higher *H. pylori* infection rates in subjects with polyps ≥ 1.0 cm compared with those having polyps < 1.0 cm among 3872 colorectal polyp patients, although the difference was not statistically significant. In contrast, Basmaci N et al^[5]^ reported a positive correlation between *H. pylori* infection rates and both polyp number and size. Research by Chen L et al^[7]^ indicated that the incidence of polyps ≥ 10 mm was significantly higher in the *H. pylori*-positive group than in the negative group. Collectively, these findings suggest that *H. pylori* infection is associated with polyp formation and growth. However, both the aforementioned studies and our own study are single-center retrospective studies and may be subject to bias. Therefore, prospective, multicenter studies are needed for further validation.

In this study, the *H. pylori* infection rates in the colorectal polyp group and the normal control group were 32.67% and 22.27%, respectively; which were lower than those reported in the literature.^[9]^ The following reasons were considered: *H. pylori* was detected by histological staining of gastric mucosal tissue sections in this study, which may yield false-negative results; the lower infection rate may be attributable to the higher socioeconomic status and better sanitary conditions in this region.

This study has several limitations. First, its single-center design requires validation through larger multicentre trials. Second, as a retrospective study, it relied on limited medical record data and did not include several potentially important factors, such as dietary habits, medication use (e.g., nonsteroidal anti-inflammatory drugs, proton pump inhibitors), socioeconomic status, and prior *H. pylori* eradication therapy. Residual confounding may have introduced bias, and future prospective studies are warranted to validate our findings. Third, *H. pylori* detection relied solely on histological staining of gastric mucosal sections. However, tissue sampling factors were not accounted for, which may have introduced classification bias. Therefore, multiple diagnostic methods should be used to determine *H. pylori* infection. Fourth, our complete-case analysis may also have introduced selection bias.

In conclusion, while *H. pylori* infection is associated with colorectal polyp development, it has limited standalone predictive value for polyp risk. Although this study used PSM to minimize bias between the 2 groups, certain important covariates could not be collected owing to study design limitations and were thus not included in the matching process; consequently, residual confounding factors might have introduced bias. Therefore, more rigorous prospective cohort studies are needed to further validate these findings.

## 5. Conclusion

Using PSM analysis, this study demonstrated that *H. pylori* infection is an independent risk factor for colorectal polyps. This association remained significant after adjusting for potential confounders.

## Author contributions

**Data curation:** Yating Gong.

**Formal analysis:** Yating Gong.

**Funding acquisition:** Yating Gong.

**Methodology:** Zhen Fan.

**Writing – original draft:** Yating Gong.

**Writing – review & editing:** Zhen Fan.

## References

[1] Fitzmaurice C Akinyemiju TF Al Lami FH. Global, regional, and national cancer incidence, mortality, years of life lost, years lived with disability, and disability-adjusted life-years for 29 cancer groups, 1990 to 2016: a systematic analysis for the global burden of disease study. JAMA Oncol. 2018;4:1553–68. 29860482 10.1001/jamaoncol.2018.2706 PMC6248091

[2] Burnett-Hartman AN Passarelli MN Adams SV. Differences in epidemiologic risk factors for colorectal adenomas and serrated polyps by lesion severity and anatomical site. Am J Epidemiol. 2013;177:625–37. 23459948 10.1093/aje/kws282 PMC3657530

[3] Hawkins NJ Ward RL. Sporadic colorectal cancers with microsatellite instability and their possible origin in hyperplastic polyps and serrated adenomas. J Natl Cancer Inst. 2001;93:1307–13. 11535705 10.1093/jnci/93.17.1307

[4] Jass JR. Hyperplastic-like polyps as precursors of microsatellite-unstable colorectal cancer. Am J Clin Pathol. 2003;119:773–5. 12817423 10.1309/UYN7-0N9W-2DVN-9ART

[5] Basmaci N Karataş A Ergin M Dumlu GS. Association between *Helicobacter pylori* infection and colorectal polyps. Medicine (Baltim). 2023;102:e35591. 10.1097/MD.0000000000035591 PMC10589529 37861565

[6] Brim H Zahaf M Laiyemo AO. Gastric *Helicobacter pylori* infection associates with an increased risk of colorectal polyps in African Americans. BMC Cancer. 2014;14:296. 24774100 10.1186/1471-2407-14-296 PMC4022546

[7] Chen L Cao RR Han J. Association of *Helicobacter pylori* infection with colorectal polyps/adenomas: a single-center cross-sectional study. Cancer Epidemiol. 2024;92:102626. 39079227 10.1016/j.canep.2024.102626

[8] Papastergiou V Karatapanis S Georgopoulos SD. *Helicobacter pylori* and colorectal neoplasia: is there a causal link? World J Gastroenterol. 2016;22:649–58. 26811614 10.3748/wjg.v22.i2.649 PMC4716066

[9] Du Y Zhu H Liu J. Consensus on eradication of *Helicobacter pylori* and prevention and control of gastric cancer in China (2019, Shanghai). J Gastroenterol Hepatol. 2020;35:624–9. 31788864 10.1111/jgh.14947

[10] Rosenbaum PR Rubin DB. The central role of the propensity score in observational studies for causal effects. Biometrika. 1983;70:41–55.

[11] Ding H Ge ZZ Fang JY. Analysis of characteristics and correlation between colorectal adenomas and *Helicobacter pylori* infection in 410 patients. Chin J Dig Endosc. 2018;35:507–10.

[12] Abbass K Gul W Beck G Markert R Akram S. Association of *Helicobacter pylori* infection with the development of colorectal polyps and colorectal carcinoma. South Med J. 2011;104:473–6. 21886044 10.1097/SMJ.0b013e31821e9009

[13] Yu R Dong WG Tian S Wang T. Clinical research progress on carcinogenesis of different pathological types of colorectal polyps. Chin General Pract. 2023;26:1790–3.

[14] Wang M Kong WJ Zhang JZ. Association of *Helicobacter pylori* infection with colorectal polyps and malignancy in China. World J Gastrointest Oncol. 2020;12:582–91. 32461789 10.4251/wjgo.v12.i5.582 PMC7235179

[15] Zhao XX Liu MH Wang RL Tian T. Effect of gender and age on the correlation between *Helicobacter pylori* and colorectal adenomatous polyps in a Chinese urban population: a single center study. Gastroenterol Res Pract. 2020;2020:1–7. 10.1155/2020/8596038 PMC7035519 32104172

[16] Austin PC. Balance diagnostics for comparing the distribution of baseline covariates between treatment groups in propensity-score matched samples. Stat Med. 2009;28:3083–107. 19757444 10.1002/sim.3697 PMC3472075

[17] Hussan H McLaughlin E Chiang C Marsano JG Lieberman D. The risk of colorectal polyps after weight loss therapy versus obesity: a propensity-matched nationwide cohort study. Cancers. 2023;15:4820. 37835515 10.3390/cancers15194820 PMC10571780

[18] Fu Z Shrubsole MJ Smalley WE. Lifestyle factors and their combined impact on the risk of colorectal polyps. Am J Epidemiol. 2012;176:766–76. 23079606 10.1093/aje/kws157 PMC3571253

[19] Qin M Wang HP Song B. Relationship between insulin resistance, serum VCAM-1, FGF19, IGF-1 and colorectal polyps. Zhonghua Zhong Liu Za Zhi. 2021;43:553–62. 34034475 10.3760/cma.j.cn112152-20210219-00146

[20] Um K Park C Yoo C Ahn Y Kim M Jeong KS. Risk factors including night shift work of colorectal polyp. Ann Occup Environ Med. 2020;32:e26. 32802342 10.35371/aoem.2020.32.e26 PMC7406667

